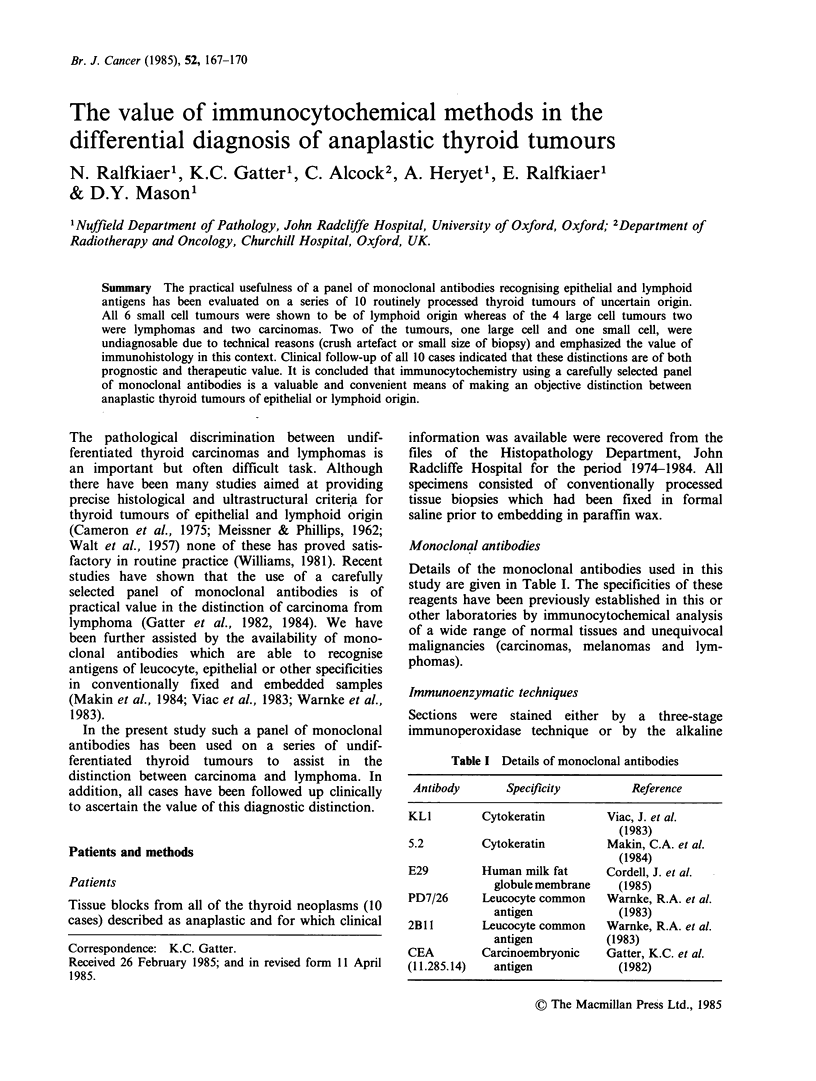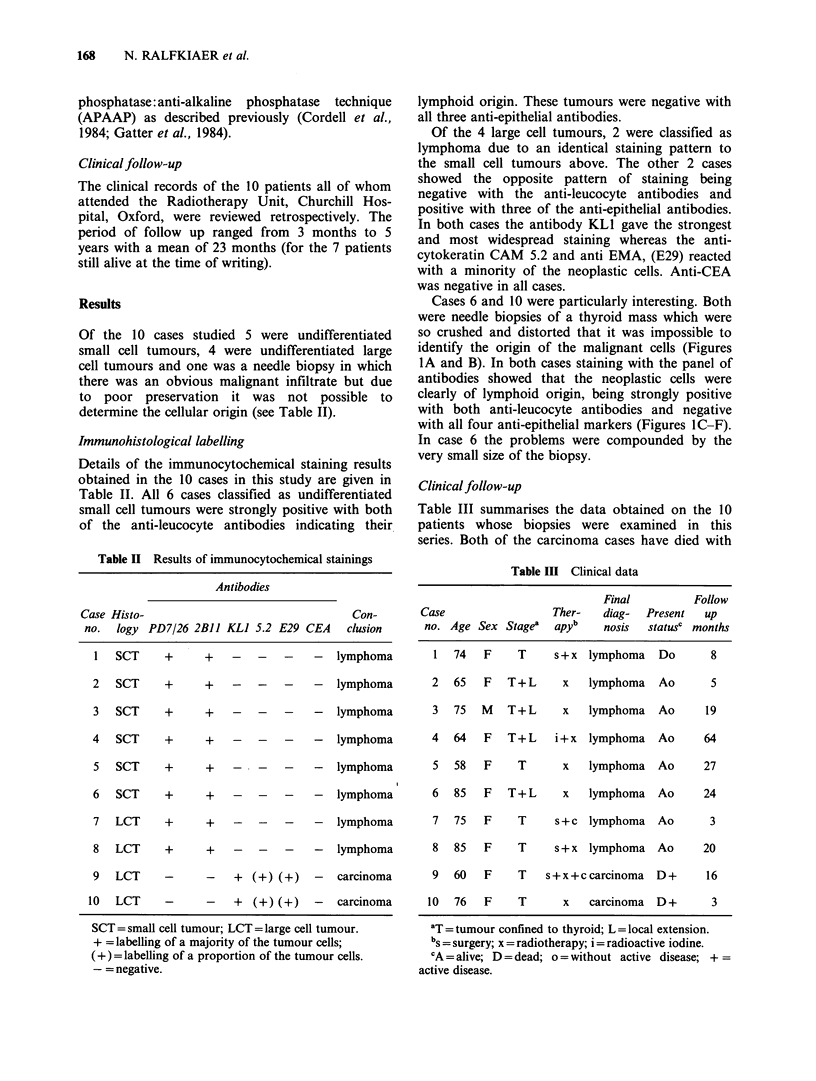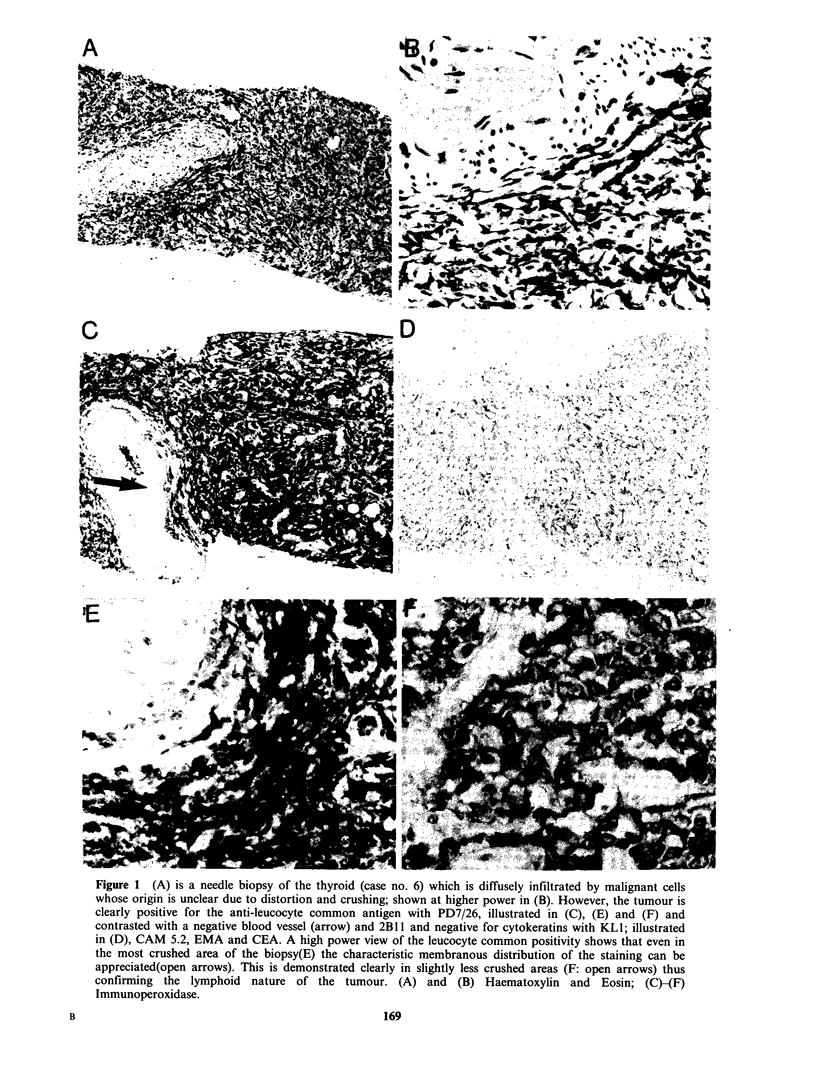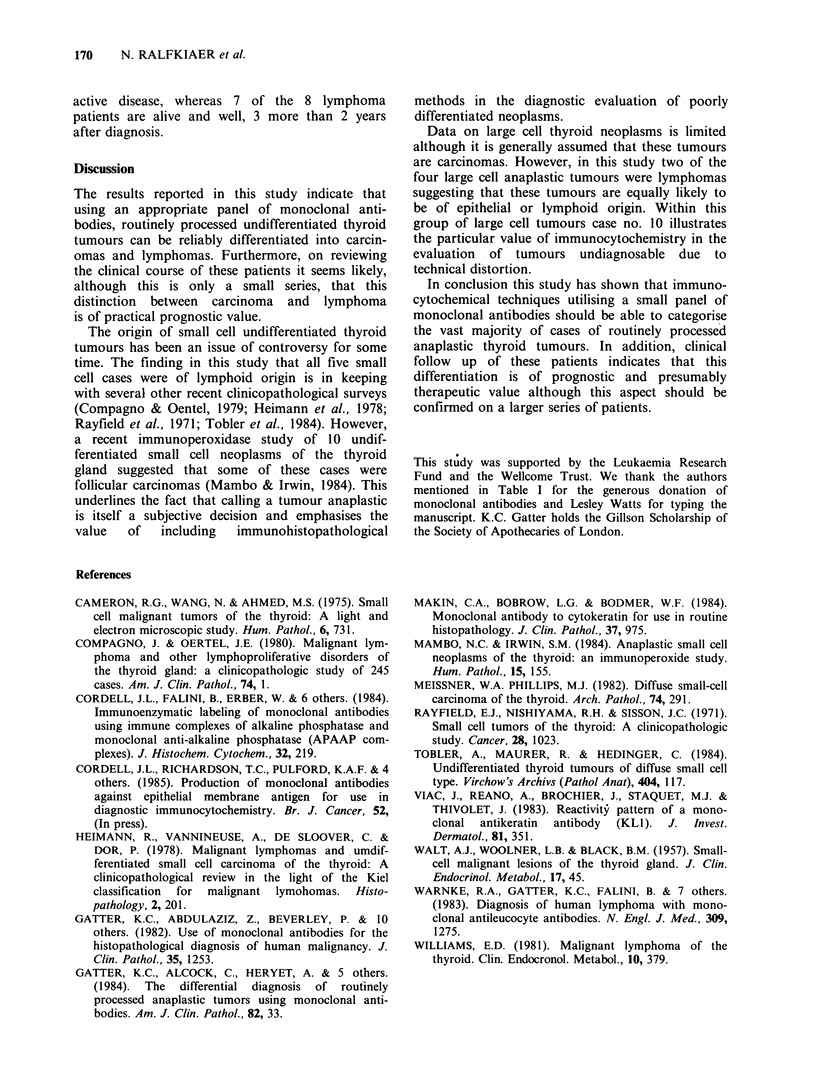# The value of immunocytochemical methods in the differential diagnosis of anaplastic thyroid tumours.

**DOI:** 10.1038/bjc.1985.173

**Published:** 1985-08

**Authors:** N. Ralfkiaer, K. C. Gatter, C. Alcock, A. Heryet, E. Ralfkiaer, D. Y. Mason

## Abstract

**Images:**


Br. J. Cancer (1985), **52**, 167–170

### The value of immunocytochemical methods in the differential diagnosis of anaplastic thyroid tumours

N. Ralfkiaer[1], K.C. Gatter[1], C. Alcock[2], A. Heryet[1], E. Ralfkiaer[1] & D.Y. Mason[1]

[1]*Nuffield Department of Pathology, John Radcliffe Hospital, University of Oxford, Oxford;* [2]*Department of Radiotherapy and Oncology, Churchill Hospital, Oxford, UK.*

**Summary** The practical usefulness of a panel of monoclonal antibodies recognising epithelial and lymphoid antigens has been evaluated on a series of 10 routinely processed thyroid tumours of uncertain origin. All 6 small cell tumours were shown to be of lymphoid origin whereas of the 4 large cell tumours two were lymphomas and two carcinomas. Two of the tumours, one large cell and one small cell, were undiagnosable due to technical reasons (crush artefact or small size of biopsy) and emphasized the value of immunohistology in this context. Clinical follow-up of all 10 cases indicated that these distinctions are of both prognostic and therapeutic value. It is concluded that immunocytochemistry using a carefully selected panel of monoclonal antibodies is a valuable and convenient means of making an objective distinction between anaplastic thyroid tumours of epithelial or lymphoid origin.

The pathological discrimination between undifferentiated thyroid carcinomas and lymphomas is an important but often difficult task. Although there have been many studies aimed at providing precise histological and ultrastructural criteria for thyroid tumours of epithelial and lymphoid origin (Cameron *et al.*, 1975; Meissner & Phillips, 1962; Walt *et al.*, 1957) none of these has proved satisfactory in routine practice (Williams, 1981). Recent studies have shown that the use of a carefully selected panel of monoclonal antibodies is of practical value in the distinction of carcinoma from lymphoma (Gatter *et al.*, 1982, 1984). We have been further assisted by the availability of monoclonal antibodies which are able to recognise antigens of leucocyte, epithelial or other specificities in conventionally fixed and embedded samples (Makin *et al.*, 1984; Viac *et al.*, 1983; Warnke *et al.*, 1983).

In the present study such a panel of monoclonal antibodies has been used on a series of undifferentiated thyroid tumours to assist in the distinction between carcinoma and lymphoma. In addition, all cases have been followed up clinically to ascertain the value of this diagnostic distinction.

### Patients and methods

### *Patients*

Tissue blocks from all of the thyroid neoplasms (10 cases) described as anaplastic and for which clinical information was available were recovered from the files of the Histopathology Department, John Radcliffe Hospital for the period 1974–1984. All specimens consisted of conventionally processed tissue biopsies which had been fixed in formal saline prior to embedding in paraffin wax.

### *Monoclonal antibodies*

Details of the monoclonal antibodies used in this study are given in Table I. The specificities of these reagents have been previously established in this or other laboratories by immunocytochemical analysis of a wide range of normal tissues and unequivocal malignancies (carcinomas, melanomas and lymphomas).

### *Immunoenzymatic techniques*

Sections were stained either by a three-stage immunoperoxidase technique or by the alkaline

**Table I** Details of monoclonal antibodies

| *Antibody* | *Specificity* | *Reference* |
|---|---|---|
| KL1 | Cytokeratin | Viac, J. *et al.* (1983) |
| 5.2 | Cytokeratin | Makin, C.A. *et al.* (1984) |
| E29 | Human milk fat globule membrane | Cordell, J. *et al.* (1985) |
| PD7/26 | Leucocyte common antigen | Warnke, R.A. *et al.* (1983) |
| 2B11 | Leucocyte common antigen | Warnke, R.A. *et al.* (1983) |
| CEA (11.285.14) | Carcinoembryonic antigen | Gatter, K.C. *et al.* (1982) |

Correspondence: K.C. Gatter.
Received 26 February 1985; and in revised form 11 April 1985.

© The Macmillan Press Ltd., 1985

phosphatase:anti-alkaline phosphatase technique (APAAP) as described previously (Cordell *et al.*, 1984; Gatter *et al.*, 1984).

*Clinical follow-up*

The clinical records of the 10 patients all of whom attended the Radiotherapy Unit, Churchill Hospital, Oxford, were reviewed retrospectively. The period of follow up ranged from 3 months to 5 years with a mean of 23 months (for the 7 patients still alive at the time of writing).

### Results

Of the 10 cases studied 5 were undifferentiated small cell tumours, 4 were undifferentiated large cell tumours and one was a needle biopsy in which there was an obvious malignant infiltrate but due to poor preservation it was not possible to determine the cellular origin (see Table II).

*Immunohistological labelling*

Details of the immunocytochemical staining results obtained in the 10 cases in this study are given in Table II. All 6 cases classified as undifferentiated small cell tumours were strongly positive with both of the anti-leucocyte antibodies indicating their lymphoid origin. These tumours were negative with all three anti-epithelial antibodies.

Of the 4 large cell tumours, 2 were classified as lymphoma due to an identical staining pattern to the small cell tumours above. The other 2 cases showed the opposite pattern of staining being negative with the anti-leucocyte antibodies and positive with three of the anti-epithelial antibodies. In both cases the antibody KL1 gave the strongest and most widespread staining whereas the anti-cytokeratin CAM 5.2 and anti EMA, (E29) reacted with a minority of the neoplastic cells. Anti-CEA was negative in all cases.

Cases 6 and 10 were particularly interesting. Both were needle biopsies of a thyroid mass which were so crushed and distorted that it was impossible to identify the origin of the malignant cells (Figures 1A and B). In both cases staining with the panel of antibodies showed that the neoplastic cells were clearly of lymphoid origin, being strongly positive with both anti-leucocyte antibodies and negative with all four anti-epithelial markers (Figures 1C–F). In case 6 the problems were compounded by the very small size of the biopsy.

*Clinical follow-up*

Table III summarises the data obtained on the 10 patients whose biopsies were examined in this series. Both of the carcinoma cases have died with

**Table II** Results of immunocytochemical stainings

| | | Antibodies | | | | | | |
|---|---|---|---|---|---|---|---|---|
| *Case no.* | *Histology* | *PD7/26* | *2B11* | *KL1* | *5.2* | *E29* | *CEA* | *Conclusion* |
| 1 | SCT | + | + | – | – | – | – | lymphoma |
| 2 | SCT | + | + | – | – | – | – | lymphoma |
| 3 | SCT | + | + | – | – | – | – | lymphoma |
| 4 | SCT | + | + | – | – | – | – | lymphoma |
| 5 | SCT | + | + | – | – | – | – | lymphoma |
| 6 | SCT | + | + | – | – | – | – | lymphoma |
| 7 | LCT | + | + | – | – | – | – | lymphoma |
| 8 | LCT | + | + | – | – | – | – | lymphoma |
| 9 | LCT | – | – | + | (+) | (+) | – | carcinoma |
| 10 | LCT | – | – | + | (+) | (+) | – | carcinoma |

SCT = small cell tumour; LCT = large cell tumour.
+ = labelling of a majority of the tumour cells;
(+) = labelling of a proportion of the tumour cells.
– = negative.

**Table III** Clinical data

| *Case no.* | *Age* | *Sex* | *Stage*[a] | *Therapy*[b] | *Final diagnosis* | *Present status*[c] | *Follow up months* |
|---|---|---|---|---|---|---|---|
| 1 | 74 | F | T | s+x | lymphoma | Do | 8 |
| 2 | 65 | F | T+L | x | lymphoma | Ao | 5 |
| 3 | 75 | M | T+L | x | lymphoma | Ao | 19 |
| 4 | 64 | F | T+L | i+x | lymphoma | Ao | 64 |
| 5 | 58 | F | T | x | lymphoma | Ao | 27 |
| 6 | 85 | F | T+L | x | lymphoma | Ao | 24 |
| 7 | 75 | F | T | s+c | lymphoma | Ao | 3 |
| 8 | 85 | F | T | s+x | lymphoma | Ao | 20 |
| 9 | 60 | F | T | s+x+c | carcinoma | D+ | 16 |
| 10 | 76 | F | T | x | carcinoma | D+ | 3 |

[a]T = tumour confined to thyroid; L = local extension.
[b]s = surgery; x = radiotherapy; i = radioactive iodine.
[c]A = alive; D = dead; o = without active disease; + = active disease.

**Figure 1** (A) is a needle biopsy of the thyroid (case no. 6) which is diffusely infiltrated by malignant cells whose origin is unclear due to distortion and crushing; shown at higher power in (B). However, the tumour is clearly positive for the anti-leucocyte common antigen with PD7/26, illustrated in (C), (E) and (F) and contrasted with a negative blood vessel (arrow) and 2B11 and negative for cytokeratins with KL1; illustrated in (D), CAM 5.2, EMA and CEA. A high power view of the leucocyte common positivity shows that even in the most crushed area of the biopsy(E) the characteristic membranous distribution of the staining can be appreciated(open arrows). This is demonstrated clearly in slightly less crushed areas (F: open arrows) thus confirming the lymphoid nature of the tumour. (A) and (B) Haematoxylin and Eosin; (C)–(F) Immunoperoxidase.

active disease, whereas 7 of the 8 lymphoma patients are alive and well, 3 more than 2 years after diagnosis.

### Discussion

The results reported in this study indicate that using an appropriate panel of monoclonal antibodies, routinely processed undifferentiated thyroid tumours can be reliably differentiated into carcinomas and lymphomas. Furthermore, on reviewing the clinical course of these patients it seems likely, although this is only a small series, that this distinction between carcinoma and lymphoma is of practical prognostic value.

The origin of small cell undifferentiated thyroid tumours has been an issue of controversy for some time. The finding in this study that all five small cell cases were of lymphoid origin is in keeping with several other recent clinicopathological surveys (Compagno & Oentel, 1979; Heimann *et al.*, 1978; Rayfield *et al.*, 1971; Tobler *et al.*, 1984). However, a recent immunoperoxidase study of 10 undifferentiated small cell neoplasms of the thyroid gland suggested that some of these cases were follicular carcinomas (Mambo & Irwin, 1984). This underlines the fact that calling a tumour anaplastic is itself a subjective decision and emphasises the value of including immunohistopathological methods in the diagnostic evaluation of poorly differentiated neoplasms.

Data on large cell thyroid neoplasms is limited although it is generally assumed that these tumours are carcinomas. However, in this study two of the four large cell anaplastic tumours were lymphomas suggesting that these tumours are equally likely to be of epithelial or lymphoid origin. Within this group of large cell tumours case no. 10 illustrates the particular value of immunocytochemistry in the evaluation of tumours undiagnosable due to technical distortion.

In conclusion this study has shown that immunocytochemical techniques utilising a small panel of monoclonal antibodies should be able to categorise the vast majority of cases of routinely processed anaplastic thyroid tumours. In addition, clinical follow up of these patients indicates that this differentiation is of prognostic and presumably therapeutic value although this aspect should be confirmed on a larger series of patients.

This study was supported by the Leukaemia Research Fund and the Wellcome Trust. We thank the authors mentioned in Table I for the generous donation of monoclonal antibodies and Lesley Watts for typing the manuscript. K.C. Gatter holds the Gillson Scholarship of the Society of Apothecaries of London.

### References

CAMERON, R.G., WANG, N. & AHMED, M.S. (1975). Small cell malignant tumors of the thyroid: A light and electron microscopic study. *Hum. Pathol.*, **6**, 731.

COMPAGNO, J. & OERTEL, J.E. (1980). Malignant lymphoma and other lymphoproliferative disorders of the thyroid gland: a clinicopathologic study of 245 cases. *Am. J. Clin. Pathol.*, **74**, 1.

CORDELL, J.L., FALINI, B., ERBER, W. & 6 others. (1984). Immunoenzymatic labeling of monoclonal antibodies using immune complexes of alkaline phosphatase and monoclonal anti-alkaline phosphatase (APAAP complexes). *J. Histochem. Cytochem.*, **32**, 219.

CORDELL, J.L., RICHARDSON, T.C., PULFORD, K.A.F. & 4 others. (1985). Production of monoclonal antibodies against epithelial membrane antigen for use in diagnostic immunocytochemistry. *Br. J. Cancer*, **52**, (In press).

HEIMANN, R., VANNINEUSE, A., DE SLOOVER, C. & DOR, P. (1978). Malignant lymphomas and umdifferentiated small cell carcinoma of the thyroid: A clinicopathological review in the light of the Kiel classification for malignant lymohomas. *Histopathology*, **2**, 201.

GATTER, K.C., ABDULAZIZ, Z., BEVERLEY, P. & 10 others. (1982). Use of monoclonal antibodies for the histopathological diagnosis of human malignancy. *J. Clin. Pathol.*, **35**, 1253.

GATTER, K.C., ALCOCK, C., HERYET, A. & 5 others. (1984). The differential diagnosis of routinely processed anaplastic tumors using monoclonal antibodies. *Am. J. Clin. Pathol.*, **82**, 33.

MAKIN, C.A., BOBROW, L.G. & BODMER, W.F. (1984). Monoclonal antibody to cytokeratin for use in routine histopathology. *J. Clin. Pathol.*, **37**, 975.

MAMBO, N.C. & IRWIN, S.M. (1984). Anaplastic small cell neoplasms of the thyroid: an immunoperoxide study. *Hum. Pathol.*, **15**, 155.

MEISSNER, W.A. PHILLIPS, M.J. (1982). Diffuse small-cell carcinoma of the thyroid. *Arch. Pathol.*, **74**, 291.

RAYFIELD, E.J., NISHIYAMA, R.H. & SISSON, J.C. (1971). Small cell tumors of the thyroid: A clinicopathologic study. *Cancer*, **28**, 1023.

TOBLER, A., MAURER, R. & HEDINGER, C. (1984). Undifferentiated thyroid tumours of diffuse small cell type. *Virchow's Archivs* (*Pathol Anat*), **404**, 117.

VIAC, J., REANO, A., BROCHIER, J., STAQUET, M.J. & THIVOLET, J. (1983). Reactivity pattern of a monoclonal antikeratin antibody (KL1). *J. Invest. Dermatol.*, **81**, 351.

WALT, A.J., WOOLNER, L.B. & BLACK, B.M. (1957). Small-cell malignant lesions of the thyroid gland. *J. Clin. Endocrinol. Metabol.*, **17**, 45.

WARNKE, R.A., GATTER, K.C., FALINI, B. & 7 others. (1983). Diagnosis of human lymphoma with monoclonal antileucocyte antibodies. *N. Engl. J. Med.*, **309**, 1275.

WILLIAMS, E.D. (1981). Malignant lymphoma of the thyroid. Clin. Endocronol. Metabol., **10**, 379.